# Effect of gait speed on post-stroke step length and anterior ground reaction force relative to individualized predicted values

**DOI:** 10.21203/rs.3.rs-6506637/v1

**Published:** 2025-05-30

**Authors:** Sarah A Kettlety, Maryana Bonilla Yanez, Christina K Holl, Jan Stenum, Ryan T Roemmich, Kristan A Leech

**Affiliations:** University of Southern California; University of Southern California; University of Southern California; Johns Hopkins University; Kennedy Krieger Institute; University of Southern California

**Keywords:** Stroke, gait, anterior ground reaction force, step length, fast walking

## Abstract

**Background.:**

Training at faster gait speeds is recommended to improve activity limitations in adults with stroke. Walking faster can also increase step length and anterior ground reaction force (AGRF) relative to post-stroke habitual walking patterns. Recent work has developed a prediction equation that utilizes individual characteristics to predict a neurotypical or pre-stroke step length and AGRF value for an individual. However, it is unclear how the predicted individualized step length and AGRF values from this prediction equation relate to actual step length and AGRF values, and how the relationship between these values may change with gait speed. We aimed to understand the effect of gait speed on post-stroke step length and AGRF relative to a predicted individualized step length and AGRF value and to understand whether step length asymmetry direction (i.e., whether the paretic or non-paretic step was longer) impacted this relationship. We hypothesized that the difference between the predicted individualized values and actual values would decrease as speed increased for both step length and AGRF.

**Methods.:**

Participants post-stroke walked on a treadmill at two or three speeds. We recorded AGRF and step length values. We then calculated an individualized predicted step length and AGRF value using a previously developed prediction equation. We fit two linear mixed effects models, the first with step length as the outcome and the second with AGRF as the outcome. Both models included fixed effects for gait speed, limb, and speed by limb interaction. We then repeated the same analyses, splitting the participants by those who took longer paretic steps and those who took longer non-paretic steps.

**Results.:**

We found that walking faster did not impact the difference between actual and predicted step lengths, regardless of which limb took a longer step. Walking faster increased the difference between paretic AGRF and the predicted AGRF value, specifically for individuals who took longer paretic steps at their self-selected gait speed.

**Conclusions.:**

Fast walking alone does not improve AGRF to the level of a predicted individualized value for individuals who took longer paretic steps. Fast walking may need to be paired with another intervention to increase paretic AGRF.

## INTRODUCTION

Walking activity limitations and biomechanical impairments are common post-stroke [1]. Activity limitations, including reduced gait speed and distance, are associated with reduced community ambulation and quality of life [2]. Biomechanical impairments, such as step length asymmetry (SLA) and reduced peak anterior ground reaction force (AGRF), are associated with balance [3], fall risk [4–6], and hemiparetic severity [7–9]. Improving both activity limitations and biomechanical gait impairments are top rehabilitation goals for adults with stroke [10].

To improve post-stroke activity limitations, high-aerobic intensity gait training, achieved by walking at faster speeds, is recommended [11, 12]. Although high-aerobic intensity gait training does not explicitly prioritize reducing biomechanical impairments, walking faster can improve certain biomechanical impairments. Walking faster increases step length [13–15], decreases SLA [13, 16], and increases AGRF [17] relative to post-stroke habitual gait patterns. However, comparing an individual’s biomechanical values only to their habitual gait pattern may mask any additional capacity to change their gait biomechanics beyond their habitual gait pattern. Comparing post-stroke biomechanical values to a neurotypical value may highlight additional deficits that still exist even with speed-dependent improvements in gait biomechanics. Therefore, it may be necessary to include speed-matched neurotypical reference data for comparison when examining post-stroke gait biomechanics. Our recent work found that walking faster reduced the differences in SLA and trailing limb angle between speed-matched neurotypical adults and adults with stroke [16]. However, it is unclear how post-stroke step length and AGRF values differ from those of a neurotypical adult across gait speeds.

Individual factors other than gait speed, such as leg length, mass, sex, and age, are related to step length and AGRF [18]. Collecting neurotypical control data for comparison that matches gait speed and these individual characteristics is not feasible. To reduce the data collection burden and establish personalized step length and AGRF values, we recently developed a prediction model that utilizes easily obtainable data (i.e., gait speed, leg length, mass, sex, and age) to predict a neurotypical (or, theoretically, pre-stroke) step length and AGRF value for an individual [18]. However, it is unclear how predicted individualized step length and AGRF values obtained from this prediction model relate to post-stroke habitual walking patterns and how the relationship between the predicted individualized values and actual values may change with gait speed.

We aimed to understand the effect of gait speed on post-stroke step length and AGRF relative to a predicted individualized step length and AGRF value. Because of previous work demonstrating that SLA and trailing limb angle become more similar to neurotypical adults at faster speeds [16], we hypothesized that the difference between the predicted individualized values and actual values would decrease as speed increased for both step length and AGRF. Additionally, there is evidence that the SLA direction (i.e., which limb takes the longer step) is related to AGRF, where those who take longer paretic steps have reduced AGRF compared to those who take longer non-paretic steps [7, 19, 20]. However, it is unclear if speed-dependent changes in step length or AGRF may differ based on which limb takes the longer step. Therefore, as a sub-analysis, we wanted to understand the impact of SLA direction on the relationship between predicted individualized values and actual step length and AGRF across gait speeds.

## METHODS

We included data from participants post-stroke walking on a treadmill from two separate datasets. Detailed inclusion and exclusion criteria for each dataset are included in the Supplemental Methods (see Additional file 1).

The first dataset consisted of 32 adults post-stroke. Participants walked on a treadmill at three speeds: self-selected, slow, and fast. Three participants only walked at slow and self-selected speeds. Self-selected and fast gait speeds were determined using the ten-meter walk test, where participants were asked to walk at a speed that felt comfortable and the fastest speed that felt comfortable. The self-selected and fast ten-meter walk tests were completed twice, and treadmill speed was determined by averaging the speed across the two trials. The slow treadmill speed was set as 50% of the self-selected speed. However, the treadmill speed was adjusted during the beginning of some trials to ensure the participant’s safety. Participants walked for approximately 120 seconds at each speed. Kinematic data were recorded at 100 Hz using a ten-camera motion capture system (Vicon, Oxford, UK). Markers were placed bilaterally over the second and fifth metatarsal heads, calcaneus, medial and lateral malleoli, shank, medial and lateral femoral epicondyles, thigh, greater trochanter, and hip. Kinetic data were recorded at 1000 Hz using an instrumented dual-belt treadmill (Motek, Amsterdam, NL). These procedures were approved by the Johns Hopkins University Institutional Review Board. All participants provided informed consent before participating.

The second dataset consisted of 22 adults post-stroke. Participants walked on a treadmill at three speeds: self-selected, slow, and fast. The self-selected and fast speeds were determined using a custom staircase algorithm [21]. We then calculated the ratio of self-selected and fast walking speeds and multiplied it by the self-selected speed to get the slow speed. Participants walked for approximately 180 seconds at each speed. Kinematic data were recorded at 100 Hz using a ten-camera motion capture system (Qualisys AB, Goteborg, Sweden). Markers were placed bilaterally on the iliac crest, greater trochanter, lateral femoral epicondyle, lateral malleolus, and fifth metatarsal head. Kinetic data were collected at 1000 Hz using an instrumented dual-belt treadmill (Bertec Corporation, Columbus, OH, USA). Participants wore a harness for safety; however, the harness did not provide body weight support. These procedures were approved by the University of Southern California Institutional Review Board. All participants provided informed consent before participating.

### Data analysis

We analyzed kinematic and kinetic data using MATLAB R2022b. Data were lowpass filtered with a cutoff frequency of 6 Hz (kinematic data) and 20 Hz (kinetic data) [22]. For the AGRF analysis, we identified foot-strike as the point at which the vertical ground reaction force reached 100 N, and toe-off at a force less than 100 N. For the step length analysis, foot-strike and toe-off were identified as the most anterior and posterior positions of the lateral malleoli markers, respectively.

For both datasets, we extracted data from 90–120 seconds of each trial to be analyzed. However, eleven participants had inconsistent force plate data during this bin (i.e., they stepped on both force plates simultaneously); therefore, we shifted the bin back 30 seconds (i.e., from 60–90 seconds) for these participants to ensure clean kinetic data.

Peak anterior ground reaction force was defined as the maximum anterior ground reaction force between each foot-strike and toe-off. Step length was defined as the fore-aft difference between the right and left lateral malleoli markers at foot-strike. We used the average step length and average AGRF for the analyses.

Using prediction models that were previously developed using neurotypical control data [18], we calculated an AGRF and step length predicted individualized value for each participant based on their speed, age, mass, and leg length. Of note, the predicted individualized value is a single value that will be compared to both the paretic and non-paretic limbs. We calculated leg length as the vertical distance between the right greater trochanter and the right lateral malleoli at mid-stance. Before calculating predicted AGRF and step length, we excluded trials if the speed, AGRF, or step length was outside of the range of data used to train the prediction models (i.e., speed ≤ 0.36 m/s, AGRF ≤ 18.2 N, step length ≤ 0.32 m).

### Statistical analysis

All statistical analyses were performed in R version 4.4.1 [23]. We fit two separate linear mixed-effects models using the lme4 package [24]. The outcome for the first model was step length, and the outcome for the second model was AGRF. Both models had fixed effects for centered gait speed, limb, and centered speed by limb interaction, with a random intercept for participant. Centered gait speed was calculated by subtracting the mean gait speed from all gait speed values, allowing us to interpret the limb coefficient at the sample’s average gait speed. The limb variable had three levels: the paretic limb, the non-paretic limb, and the predicted individualized value. If the interaction term was significant, we used the emtrends function [25] to understand which slopes were significantly different. All linear regression assumptions were checked using the performance package [26]. The step length model met all linear regression assumptions. The AGRF model violated linear regression assumptions; therefore, we ran a robust mixed-effects model to account for those violations [27]. To obtain estimates for 95% confidence intervals for the AGRF robust model coefficients, we performed a non-parametric bootstrap with 1000 iterations [28, 29].

For our sub-analysis, we wanted to understand the impact of SLA direction on the relationship between predicted individualized step length and AGRF values and actual step length and AGRF across gait speeds. To calculate SLA, we took the difference between the paretic and non-paretic step lengths and divided it by the sum of the paretic and non-paretic step lengths. If SLA was positive, the participant took longer paretic step lengths. We performed the same analyses described above, separating the individuals who took longer paretic steps and those who took longer non-paretic steps at their self-selected speed. For the AGRF sub-analysis only, we fit robust linear mixed effects models to account for the presence of outliers [27]. To obtain estimates for 95% confidence intervals for the robust model coefficients, we performed a non-parametric bootstrap with 1000 iterations [28, 29]. For the sub-analyses only, we excluded participants with an SLA magnitude less than or equal to 0.02, which is the bounds of the 95% confidence interval for the neurotypical adult data used to train the prediction model.

## RESULTS

We included 31 participants in the step length analysis and 32 participants in the AGRF analysis. When splitting the data by SLA direction, twelve participants with longer non-paretic steps and nine with longer paretic steps were included in the step length and AGRF sub-analyses. Each participant included had data for two or three gait speeds. Detailed reasons for participant exclusion are included in Supplemental Table 1 (see Additional file 1). Clinical demographics are included in Table 1.

### Step length

**Figure 1:**
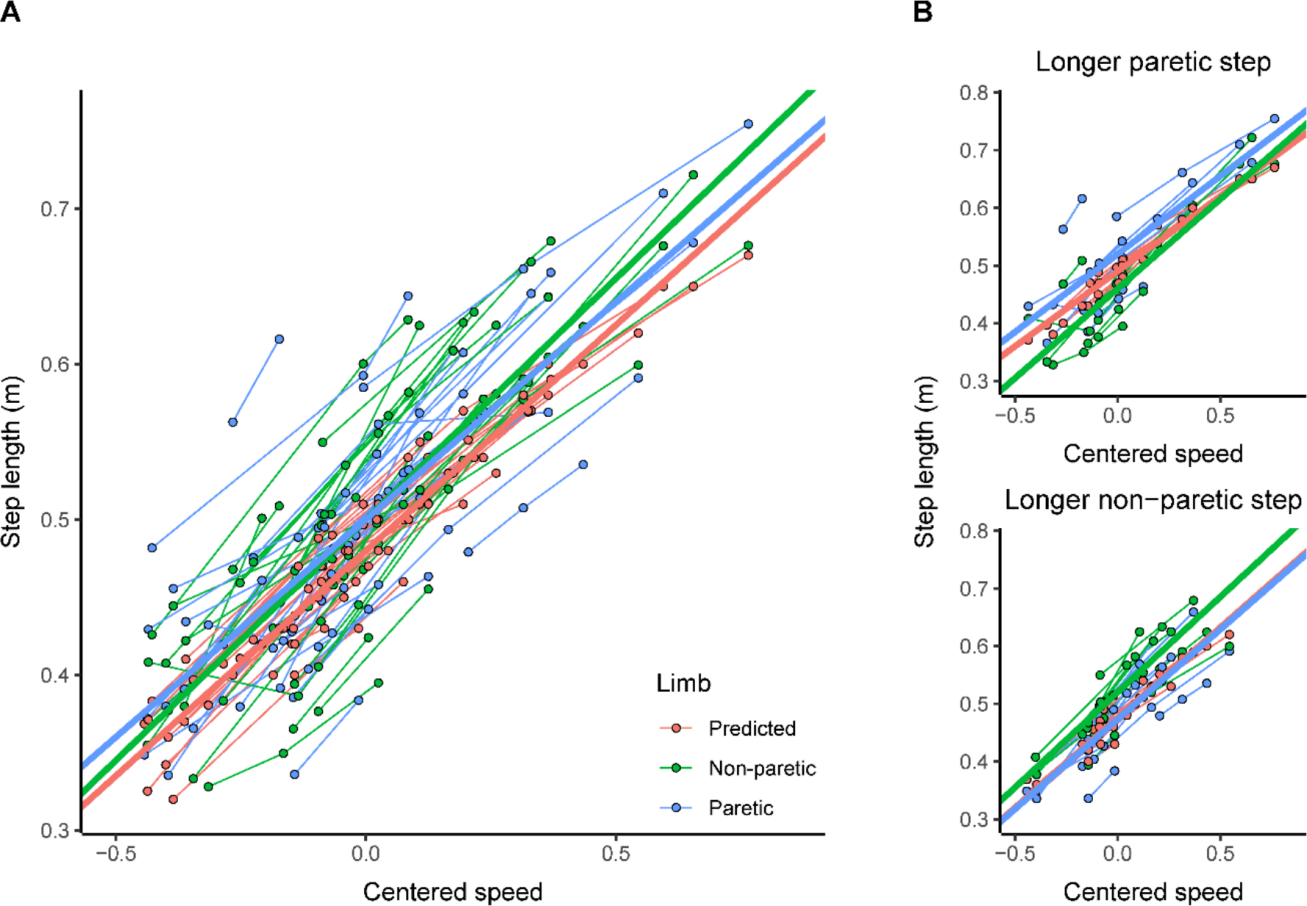
Step length across centered gait speeds. Each data point represents data from a trial for an individual participant. The thicker lines represent the fits from the mixed-effects models. Both the data and model fits are colored by limb. **A)**Step length across centered gait speeds for all participants. **B)** Step length across centered gait speeds, split by participants who took longer paretic steps (top panel) and participants who took longer non-paretic steps (bottom panel).

Across all participants, both paretic and non-paretic limbs took slightly longer steps than the predicted step length value (Fig. 1A; paretic limb: β(SE) = 0.02(0.006), 95% CI [0.01, 0.03], p = 0.0002; non-paretic limb: β(SE) = 0.03(0.006), 95% CI [0.01, 0.03], p = 0.0002). The predicted step length value increased with increased speed (β(SE) = 0.29(0.02), 95% CI [0.26, 0.32], p < 0.0001). The slopes between limbs did not differ (centered speed by non-paretic limb: β(SE) = 0.02(0.02), 95% CI [−0.02, 0.06], p = 0.39; centered speed by paretic limb: β(SE) = −0.01(0.02), 95% CI [−0.05, 0.03], p = 0.71), suggesting that changing gait speed does not affect the differences between paretic and non-paretic step lengths and the predicted step length value.

In participants who took longer paretic steps at their self-selected speed, the paretic limb took longer steps than the predicted step length value (Fig. 1B, top panel; β(SE) = 0.03(0.01), 95% CI [0.01, 0.05], p < 0.0001), and the non-paretic limb took shorter steps compared to the predicted step length value (β(SE) = −0.03(0.01), 95% CI [−0.04, −0.005], p = 0.02). We found no significant difference between non-paretic or paretic limb slopes and the predicted slope (paretic limb by centered speed: β(SE) = 0.01(0.03), 95% CI [−0.05, 0.07], p = 0.70; non-paretic limb: β(SE) = 0.05(0.03), 95% CI [−0.01, 0.11], p = 0.12). This suggests that changing gait speed does not affect the difference between paretic and non-paretic step lengths and the predicted step length value in participants who took longer paretic steps.

In participants who took longer non-paretic steps at their self-selected speed, the non-paretic limb took significantly longer steps compared to the predicted step length value (Fig. 1B, bottom panel; (β(SE) = 0.04(0.007), 95% CI [0.03, 0.05], p < 0.0001). However, the paretic limb was not significantly different from the predicted step length value (β(SE) = −0.003(0.007), 95% CI [−0.02, 0.01], p = 0.70). We found no significant difference between non-paretic or paretic limb slopes and the predicted slope (paretic limb by centered speed: β(SE) = −0.001(0.03), 95% CI [−0.06, 0.06], p = 0.97; non-paretic limb: β(SE) = 0.02(0.03), 95% CI [−0.04, 0.08], p = 0.58).

### Anterior ground reaction force

**Figure 2:**
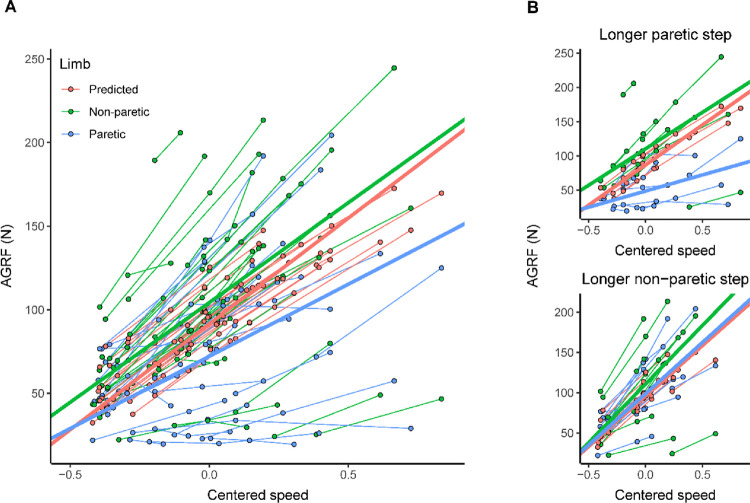
Anterior ground reaction force across centered gait speeds. Each data point represents data from a trial for an individual participant. The thicker lines represent the fits from the mixed-effects models. Both the data and model fits are colored by limb. **A)** Anterior ground reaction force across centered gait speeds for all participants. **B)** Anterior ground reaction force across centered gait speeds, split by participants who took longer paretic steps (top panel) and participants who took longer non-paretic steps (bottom panel). Abbreviations: AGRF, anterior ground reaction force.

Across all participants, the paretic limb had reduced AGRF compared to the predicted AGRF value (Fig. 2A, β(SE) = −19.4(3.2), 95% CI [−29.1, −9.4], p < 0.0001). Conversely, the non-paretic limb had higher AGRF compared to the predicted AGRF value (β(SE) = 12.8(3.2), 95% CI [3.8, 21.0], p < 0.0001). The predicted AGRF value increased as gait speed increased (β(SE) = 126.6(8.5), 95% CI [115.2, 145.0], p < 0.0001). The non-paretic limb slope was not significantly different from the predicted slope (centered speed by non-paretic limb interaction: β(SE) = −7.2(11.0), 95% CI [−50.2, 27.2], p = 0.79). The paretic limb slope was significantly less steep than the predicted slope (centered speed by paretic limb interaction: β(SE) = −40.2(11.0), 95% CI [−82.5, −2.8], p = 0.0008), suggesting that as gait speed increases, participants’ paretic AGRF moved further away from the predicted AGRF value.

In participants who took longer paretic steps at their self-selected speed, the paretic limb had significantly reduced AGRF compared to the predicted AGRF value (Fig. 2B, top panel; β(SE)= −38.0(4.3), 95% CI [−49.5, −26.0], p < 0.0001). Conversely, the non-paretic limb had significantly higher AGRF compared to the predicted AGRF value (β(SE) = 23.7(4.3), 95% CI [7.4, 38.6], p < 0.0001). Only the paretic limb slope was significantly different than the predicted slope (centered speed by paretic limb interaction: β(SE) = −70.0(12.6), 95% CI [−130.2, −17.6], p < 0.0001; centered speed by non-paretic limb interaction: β(SE) = −10.6(12.1), 95% CI [−147.9, 55.3], p = 0.68). This suggests that in participants who took longer paretic steps, paretic AGRF moved further away from the predicted AGRF value as gait speed increased.

In participants who took longer non-paretic steps at their self-selected speed, the non-paretic limb had significantly higher AGRF compared to the predicted AGRF value (Fig. 2B, bottom panel; β(SE) = 14.9(4.6), 95% CI [−3.8, 24.8], p = 0.002). However, it is important to note that the 95% bootstrap confidence interval includes zero, indicating that this finding may not be robust. The paretic limb AGRF was not significantly different from the predicted AGRF value (β(SE) = 2.9(4.6), 95% CI [−8.3, 17.5], p = 0.54). There was no significant difference in either paretic or non-paretic slopes compared to the predicted slope (paretic limb by centered speed: β(SE) = 2.0(16.9), 95% CI [−36.2, 53.5], p = 0.91; non-paretic limb: β(SE) = 20.2(16.9), 95% CI [−60.5, 58.4], p = 0.24).

## DISCUSSION

We aimed to understand how altering gait speed changed the distance from a predicted individualized value for step length and AGRF in adults with stroke. We found that walking faster did not impact the distance between actual and predicted step lengths, regardless of which limb took a longer step. Walking faster increased the difference between paretic AGRF and the predicted AGRF value, specifically for individuals who took longer paretic steps at their self-selected gait speed. This suggests that fast walking alone does not change AGRF to the level of a predicted individualized value for individuals who take longer paretic steps. Fast walking may need to be paired with another intervention to increase paretic AGRF.

### Walking faster did not affect the difference between actual and predicted step lengths

Across all participants, we found a significant effect of step length, where adults with stroke took longer paretic and non-paretic steps compared to the predicted individualized value. When we split participants by which limb took the longer step, those who took longer paretic step lengths had longer paretic steps and shorter non-paretic steps compared to the predicted individualized value. For the participants with longer non-paretic steps, the non-paretic step length was significantly longer than the predicted individualized value; the paretic limb step length was not significantly different than the predicted individualized value. Walking at faster speeds did not affect the difference between paretic or non-paretic step lengths and the predicted individualized value, regardless of which limb takes the longer step. This contrasts with our previous work, which found that SLA magnitude moves closer to a speed-matched neurotypical control as gait speed increases [16]. This difference could be due to the relatively low SLA magnitude within our sample (0.04 ± 0.03). Additionally, at the average gait speed, the paretic step length was 2 cm longer than the predicted step length, and the non-paretic step length was 3 cm longer than the predicted step length. While this was a statistically significant difference, it is a relatively small difference that may be within the error of the step length measurement [30]. Since participants were close to the predicted step length at the average gait speed, they may not be able to move closer to the prediction at faster speeds.

### Fast walking increased the difference between paretic AGRF and predicted AGRF in participants who took longer paretic steps

In participants with longer paretic step lengths, we found that paretic AGRF was significantly lower than the predicted individualized value, and non-paretic AGRF was significantly higher. This is consistent with previous work, which found that adults post-stroke who take longer paretic steps rely on non-paretic AGRF to compensate for the reduced paretic AGRF [19]. In participants who took longer non-paretic steps, neither the paretic nor non-paretic AGRF differed from the predicted individualized value. This finding contrasts with previous work, which found that individuals who take longer non-paretic steps have a higher paretic AGRF compared to speed-matched neurotypical controls [19]. A potential reason for the discrepancy is that we used a prediction model that provides an individualized AGRF value (based on gait speed and individual characteristics) instead of a speed-matched neurotypical control. This could provide a different AGRF value for comparison, as leg length and mass are also significant predictors of AGRF [18].

Fast walking increased the difference between paretic AGRF and the predicted individualized value, specifically for participants who took longer paretic steps at their self-selected speed. The shallower paretic AGRF slope seemingly contrasts with our previous work, where we found that fast walking reduced trailing limb angle difference between adults with stroke and speed-matched neurotypical adults [16]. Trailing limb angle and AGRF are related [31]; however, plantar flexor moment also contributes to AGRF [32]. At self-selected speeds, adults post-stroke have lower plantar flexor moments compared to speed-matched neurotypical controls [19]. After twelve weeks of fast walking training, individuals post-stroke primarily increased AGRF by increasing trailing limb angle, with minimal changes in plantar flexor moment [33]. This suggests that when walking faster, adults with stroke may not be able to change their trailing limb angle sufficiently to compensate for reduced plantar flexor moment and, therefore, are unable to achieve a neurotypical AGRF. To achieve a neurotypical AGRF, fast walking may need to be combined with an intervention that can improve plantar flexor moment, such as AGRF biofeedback [34] or functional electrical stimulation paired with fast walking [33].

### Benefits and limitations of using prediction models to establish individualized values for post-stroke gait analysis and experimentation

The prediction equations used in this study [18] provide a standardized and individualized method for establishing neurotypical control values for individuals with gait dysfunction. These equations can also be used to set goals for various interventions (e.g., gait biofeedback) that are not limited by an individual’s habitual gait pattern and can also inform how the goal should be set for an individual (i.e., increase paretic to match non-paretic, decrease non-paretic and increase paretic, or only increase paretic). For example, participants in our study with longer paretic steps had higher non-paretic and lower paretic AGRF than the predicted individualized value. Using a goal-setting method rooted in an individual’s habitual gait pattern, such as trying to equalize the magnitude of the paretic and non-paretic limbs, may cause an individual to overshoot the neurotypical prediction. Individuals with longer non-paretic steps tend to compensate for low paretic AGRF with higher non-paretic AGRF [19]; therefore, matching the higher, compensatory non-paretic AGRF may not be beneficial.

We found that many participants had to be excluded from our analysis because their gait speed, AGRF, or step length fell outside the bounds of the prediction models (23 from step length analysis and 22 from AGRF analysis). This is a limitation of the prediction models since many adults with stroke walk at slower speeds than are represented in the model (n = 14 had at least one trial excluded due to speed).

## CONCLUSIONS

We found that walking faster did not change the relationship between actual step lengths and the predicted individualized step length value in adults with stroke. Walking faster increased the difference between paretic AGRF and the predicted individualized value, specifically for individuals who took longer paretic steps at their self-selected gait speed. This suggests that for individuals who take longer paretic steps, fast walking alone does not improve AGRF to a predicted individualized value. Fast walking may need to be paired with another intervention, such as biofeedback or functional electrical stimulation, to increase paretic AGRF.

## Figures and Tables

**Table 1:** Clinical demographics for participants who were included in at least one analysis.

ID	Analysis included	Sex	Age	LE-FM	SS SLA	Slow Speed (m/s)	SS speed (m/s)	Fast speed (m/s)
5	SL, AGRF	M	51	-	-0.04	0.45	0.90	1.12
7	SL, AGRF	M	83	-	-0.08	0.37*	0.71	0.84
8	SL, AGRF	M	53	-	0.01	-	0.49	0.65
9	SL, AGRF	M	70	-	-0.01	0.43	0.85	0.94
10	SL, AGRF	M	60	-	-0.05	0.41	0.79	0.98
11	SL, AGRF	M	56	-	-0.05	0.39*	0.77	1.22
12	SL, AGRF	M	73	-	-0.03	0.36*	0.71	0.77
13	SL, AGRF	M	63	-	-0.04	0.40*	0.79	1.07
14	SL, AGRF	F	54	-	0.09	0.32*	0.59	0.68
17	SL, AGRF	M	58	-	-0.01	0.49	0.96	-
18	AGRF	M	69	-	-0.04	0.39	0.77	-
19	SL, AGRF	M	52	-	0.07	0.39*	0.77	0.88
25	SL, AGRF	M	69	-	-0.03	0.37*	0.71	0.84
26	SL, AGRF	F	68	-	-0.02	0.34*	0.67	0.81
27	SL, AGRF	F	68	-	-0.02	0.42	0.81	1.05
29	SL, AGRF	M	70	-	-0.05	0.39*	0.77	0.94
31	SL, AGRF	M	74	-	-0.07	0.35*	0.68	0.96
32	SL, AGRF	M	56	-	-0.02	0.60	1.19	-
34	SL, AGRF	M	74	33	-0.03	0.74*	1.02	1.40
35	SL	F	51	25	0.09	0.54	0.69	0.88
38	SL, AGRF	M	45	22	0.01	0.63*	0.88	1.22
39	SL	M	52	22	-0.08	1.06	1.17	1.29
41	AGRF	M	45	19	0.13	0.40*	0.50	0.62
42	SL	M	49	21	0.01	0.24*	0.47	0.93
43	SL, AGRF	F	30	29	0.01	0.79	0.93	1.09
44	SL, AGRF	F	31	20	0.01	0.57	0.82	1.18
45	SL, AGRF	M	49	24	0.12	0.42	0.72	1.22
46	AGRF	M	64	16	-0.02	0.40	0.44	0.49
47	SL, AGRF	M	40	29	0.07	0.85*	1.17	1.62
48	SL, AGRF	F	53	22	-0.06	0.21*	0.46	1.03
50	SL, AGRF	F	23	27	0.03	0.51	0.88	1.51
51	SL, AGRF	M	58	26	0.05	0.59*	0.76	0.98
52	SL, AGRF	M	63	19	0.03	0.59*	0.71	0.86
53	AGRF	M	53	25	-	0.66	0.78	0.92
54	SL, AGRF	M	49	29	0.04	0.76	1.05	1.45

Participants with a negative SLA value are taking longer non-paretic steps.

* denotes that the data at this speed was excluded from at least one analysis. Please see Supplemental Table 1 for details. LE-FM scores were not available for dataset 1.

Abbreviations: SL, step length; AGRF, anterior ground reaction force; M, male; F, female; LE-FM, Lower-Extremity Fugl-Meyer; SS, self-selected; SLA, step length asymmetry.

## Data Availability

The datasets used in the current study are available from RTR (dataset 1) and KAL (dataset 2) upon reasonable request.

## Abbreviations

AGRF: anterior ground reaction force.
SLA: step length asymmetry.
